# Sjögren’s syndrome and Parkinson’s Disease: A bidirectional two-sample Mendelian randomization study

**DOI:** 10.1371/journal.pone.0298778

**Published:** 2024-04-03

**Authors:** Xin Cai, Zexu Jin, Shaoqin Zhang, Jiajun Liu, Zong Jiang, Fang Tang, Tianzuo Lan

**Affiliations:** 1 Department of Rheumatology, The First People’s Hospital of Guiyang, Guiyang, Guizhou Province, China; 2 Department of Dermatology, The First People’s Hospital of Guiyang, Guiyang, Guizhou Province, China; 3 Department of Rheumatology, The Second Affiliated Hospital of Guizhou University of Traditional Chinese Medicine, Guiyang, Guizhou Province, China; Wuhan Mental Health Centre, CHINA

## Abstract

**Background:**

Previous observational studies have reported an association between Sjögren’s syndrome (SS) and an increased risk of Parkinson’s Disease (PD). However, the causal relationship between these conditions remains unclear. The objective of this study was to investigate the causal impact of SS on the risk of developing PD, utilizing the Mendelian randomization (MR) approach.

**Methods:**

We conducted a bidirectional MR analysis using publicly available genome-wide association studies (GWAS) data. The primary analysis utilized the inverse-variance weighted (IVW) method. Complementary methods, such as MR-Egger regression, weighted mode, weighted median, and MR-pleiotropy residual sum and outlier (MR-PRESSO), were utilized to identify and correct for the presence of horizontal pleiotropy.

**Results:**

The IVW MR analysis revealed no significant association between SS and PD (IVW: OR = 1.00, 95% CI = 0.94–1.07, *P* = 0.95). Likewise, the reverse MR analysis did not identify any significant causal relationship between PD and SS (IVW: OR = 0.98, 95% CI = 0.85–1.12, *P* = 0.73). The results from MR-Egger regression, weighted median, and weighted mode approaches were consistent with the IVW method. Sensitivity analyses suggested that horizontal pleiotropy is unlikely to introduce bias to the causal estimates.

**Conclusion:**

This study does not provide evidence to support the assertion that SS has a conclusive impact on the risk of PD, which contradicts numerous existing observational reports. Further investigation is necessary to determine the possible mechanisms behind the associations observed in these observational studies.

## 1. Introduction

Sjögren’s syndrome (SS) is a multifaceted and diverse chronic autoimmune disease that affects multiple organ systems. Its main symptoms typically include dry mouth and dry eyes [1]. According to the strict definition given by the American-European Consensus Criteria, it is regarded as one of the most prevalent autoimmune disorders, with an estimated prevalence ranging from 0.1% to 4.8% in different populations [2]. SS can lead to damage in various organs and systems, giving rise to a wide range of complications such as lymphoma, autoimmune hepatitis, interstitial lung disease, and immune thrombocytopenia [3]. These complications impose significant economic burdens on patients, their families, and healthcare services [4]. Furthermore, SS can result in decreased physical performance, along with feelings of anxiety, depression, and fatigue, significantly impacting the overall quality of life for patients [5]. Although the precise cause of SS remains uncertain, prior studies have indicated a potential connection between the development of SS and lymphocytic infiltration as well as immunologically mediated mechanisms [6]. The infiltration of lymphocytes and immunoreactive proteins into the nervous system can lead to a variety of neurological impairments [7]. For instance, research findings indicate that approximately 31% of individuals diagnosed with SS experience nervous system impairment [8].

Parkinson’s disease (PD) is the second most common neurodegenerative disease, impacting around 1% of individuals aged 60 and older [9]. Moreover, the prevalence of PD is projected to double within the next three decades [9]. The pathophysiological hallmark of PD is the substantial depletion of dopaminergic neurons in the substantia nigra pars compacta, resulting in reduced dopamine levels in the brain [10]. Motor symptoms of PD encompass bradykinesia, resting tremor, rigidity, and postural instability, while non-motor features include constipation, sleep disturbances, cognitive decline, and depression [11]. These challenges can also pose a significant burden on society and the healthcare system [12]. Therefore, to prevent and promptly detect PD, it is essential to investigate its risk factors. Furthermore, accumulating evidence suggests the involvement of immune dysfunction in PD pathogenesis, suggesting the potential autoimmune origins of PD [13].

An increasing observation has been made regarding the association between autoimmune diseases and PD [14]. However, epidemiological studies investigating the relationship between SS and PD have produced inconsistent and contradictory findings, leading to controversy. A cohort study involving 17,028 patients with SS and 68,094 matched non-SS controls unveiled a significantly elevated prevalence of PD in the SS cohort in comparison to non-SS individuals [15]. These findings strongly indicate that SS autonomously contributes to the risk of PD. Additionally, various case-control studies have consistently demonstrated a substantial correlation between SS and an augmented risk of PD [16, 17]. Nonetheless, it is important to note that conflicting results have been reported by other studies, indicating no heightened risk of PD among SS patients [18, 19]. Based on the available evidence, inconsistent findings exist regarding the association between SS and PD. Drawing definitive conclusions about causality solely from observational designs is not feasible due to inherent limitations in cohort and case-control studies, including limited representation of diverse races, small sample sizes, and potential confounding factors and biases [20]. Currently, the existence of a causal relationship between SS and PD remains uncertain, and if such a relationship does exist, it is unclear whether it operates in a unidirectional or bidirectional manner. Given the conflicting findings from previous research, a thorough examination of the causal relationship between SS and PD is necessary to enhance the existing evidence.

Mendelian randomization (MR) analysis is a reliable method that utilizes summary data from genome-wide association studies (GWAS) to assess causal relationships in the exposure-outcome pathway [21]. The random arrangement of genetic variation during meiosis, followed by fixation after fertilization, effectively reduces residual confounding factors and addresses reverse causality [22]. This study utilized naturally allocated genetic instrumental variables (IVs) in a MR framework to simulate a randomized controlled trial using individual or summary-level data from observational studies [23]. To avoid the need for personal-level data, a widely adopted method known as two-sample MR analysis was employed, which allows for the use of GWAS summary statistics [24]. Specifically, a bidirectional two-sample MR analysis was conducted in order to examine the causal association and directionality between SS and PD, utilizing publicly available summary statistics obtained from GWAS.

## 2. Materials and methods

### 2.1 Study design

**Fig 1:**
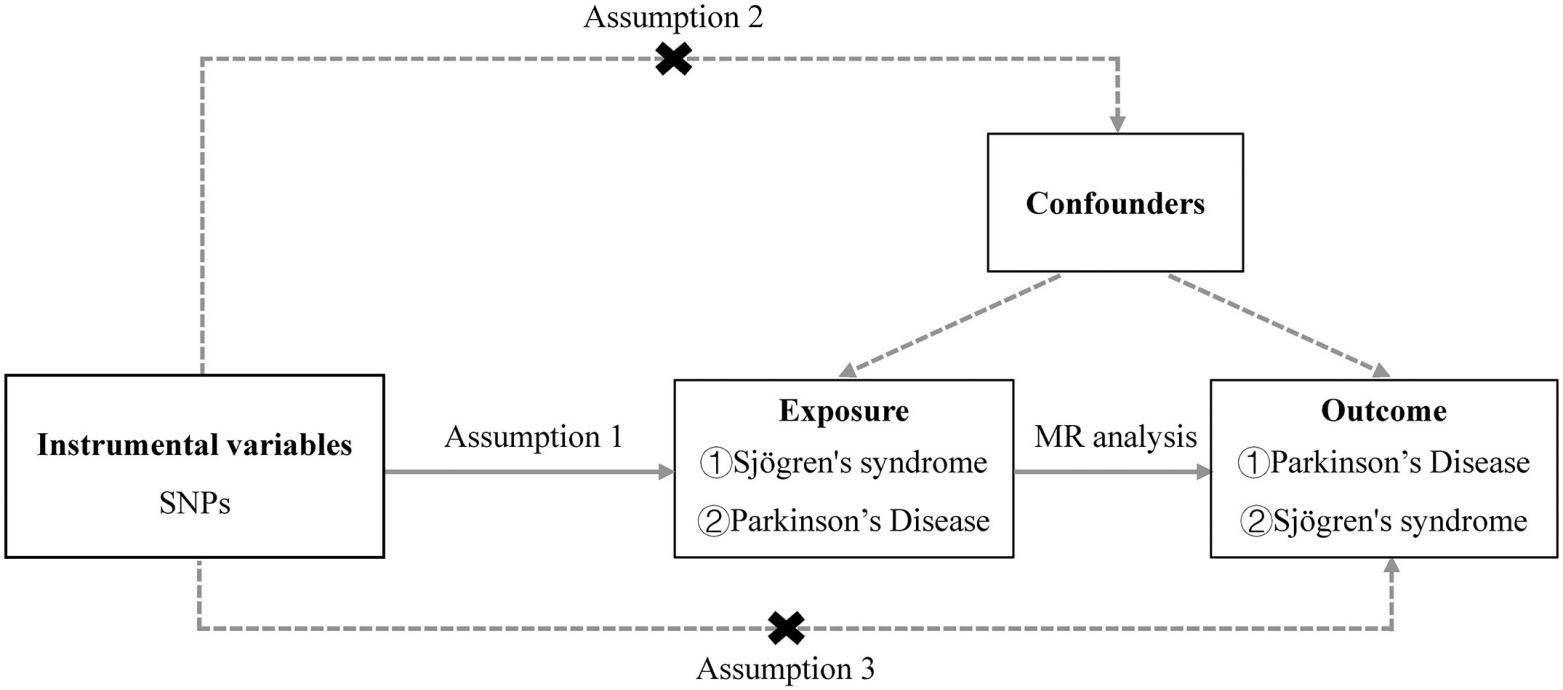
An overview of this MR study design.

We employed a two-sample bidirectional MR approach to systematically investigate the potential causal relationship between SS and PD. For instrumental variables to be considered reliable tools for causal inference in MR studies, they must satisfy three fundamental assumptions [23]. The first assumption asserts that the instrumental variable, which corresponds to genetic variation in our case, must exhibit a genuine association with the exposure to either SS or PD. The second assumption guarantees that any confounding factors potentially impacting the relationship between the exposure and the outcome will not influence the genetic variation. Furthermore, the third assumption stipulates that the genetic variation solely influences the outcome (SS or PD) through the exposure, independent of alternative pathways. Fig 1 presents an overview of the designs employed in the study. This MR study utilized previously published, publicly available large-scale GWAS datasets. Written informed consent was obtained from all individuals involved in the original GWAS study. Ethical approval and participant consent were acquired prior to the study, rendering additional approvals unnecessary for the analysis.

### 2.2 Data sources

Summary data for SS was obtained from FinnGen (https://www.finngen.fi) using the phenotype code “M13_SJOGREN” [25]. The dataset comprises 368,028 samples, with 2,495 cases and 365,533 controls. In total, 20,170,011 single nucleotide polymorphisms (SNPs) were genotyped. All participants have European ancestry. Additionally, publicly available summary statistics from the International Parkinson’s Disease Genomics Consortium (IPDGC) study [26] were employed. This dataset included 482,730 individuals, with 33,674 cases and 449,056 controls from Europe. The IPDGC summary statistics provided meta-analysis results for approximately 17,481,455 SNPs. Further details on data sources and methods can be found in the original publications.

### 2.3 Genetic instruments

To ensure the accuracy and validity of the causal relationship inferences between SS and PD, several steps were conducted to select the most appropriate IVs [27]. First, SNPs demonstrating significant associations with SS levels (*P* < 5 × 10^−8^) were chosen as IVs. Second, to address potential interpretation bias, a clumping step was performed on the selected SNPs using a clumping distance of 10,000 kb and an r^2^ threshold of < 0.001 to reduce linkage disequilibrium. Third, SNPs with a palindromic structure, which could hinder accurate estimation of the impact on outcome and exposure in MR analysis, were excluded. Additionally, the strength of genetic instruments was quantified using the F statistic, which is calculated as β^2^/se^2^. In the follow-up analysis, IVs with an F statistic greater than 10 are considered [28]. The same criteria were applied to identify IVs significantly associated with PD.

### 2.4 Statistical analysis

Before conducting MR analysis, the data should be harmonized using the previously described method to ensure consistent effect sizes for exposure and outcome among individuals with the same effect allele. In primary MR analysis, the Inverse Variance Weighted (IVW) method is employed. The IVW method relies on the assumption that either the MR assumptions are met or all SNPs included are valid instruments [29]. Additional analyses were conducted using MR-Egger, weighted mode, and weighted median methods. The aim of MR-Egger analysis is to assess the directional pleiotropy effects of the IVs on the outcome [30]. The weighted median method provides reliable causal estimates when more than 50% of the IVs offer reliable information [31]. However, compared to IVW, the power of weighted median, weighted mode, and MR-Egger methods is relatively limited, which results in wider confidence intervals (CIs). As a result, these methods were solely employed as complementary approaches in this study. Additionally, we conducted another search for SNPs in the PhenoScanner database (http://www.phenoscanner.medschl.cam.ac.uk) to eliminate potential pleiotropy by excluding previously reported SNPs associated with confounding factors. Subsequently, we performed a second round of MR analysis to investigate more direct causal associations.

We conducted statistical analysis using the R programming language (version 4.2.3). MR analysis was performed using the “TwoSampleMR” package (version 0.5.6) [32]. In addition, we employed the “MRPRESSO” package (version 1.0) to identify outliers and evaluate pleiotropy through MRPRESSO analysis [33].

### 2.5 Sensitivity analyses

Performing sensitivity analysis is crucial in order to ensure the validity and reliability of the MR results. In our research, we employed Cochran’s Q statistic to evaluate the degree of heterogeneity among each SNPs. Specifically, we deemed significant heterogeneity to exist when Cochran’s Q statistic yielded a *P*-value below 0.05 [34]. Horizontal pleiotropy was assessed by examining the intercept of the MR-Egger regression model, whereby its presence was indicated by a P-value below 0.05 [30]. Furthermore, we employed the MR-Pleiotropy Residual Sum and Outlier (MR-PRESSO) techniques to identify potential outliers and cases of horizontal pleiotropy, as denoted by a global *P*-value less than 0.05 [33]. Any identified outliers were subsequently excluded from the analysis to enhance the accuracy of our adjusted estimates. Lastly, the stability of the MR estimates was evaluated through leave-one-out analysis, in which individual SNPs were systematically excluded. Publication bias was evaluated through the examination of funnel plots for symmetry and the assessment of potential directional pleiotropy. The effect estimates between genetic variants and SS or PD were assessed using forest plots, and the combined effects were calculated using the MR-Egger regression with IVW. Additionally, to mitigate the potential for reverse causation, further MR and sensitivity analyses were conducted by switching the outcome and exposure variables.

## 3. Results

MR analysis necessitates satisfying three fundamental assumptions. In our study, we identified six SNPs closely associated with SS and twenty-one SNPs closely associated with PD from GWAS summary data. Thus, fulfilling the initial assumption that IVs are strongly associated with the exposure. Through the evaluation and screening of linkage disequilibrium between SNPs, and the utilization of PhenoScanner to exclude additional confounding factors, we satisfy the second assumption that IVs are independent of any confounding factors. By employing sensitivity analysis and heterogeneity analysis to eliminate potential pleiotropic effects, we guarantee trustworthy and valid results for the MR analysis, without any significant evidence of pleiotropic effects. Moreover, this approach satisfies the third assumption that IVs solely impact the outcome via the exposure.

### 3.1 Causal effects of SS on PD

**Fig 2:**
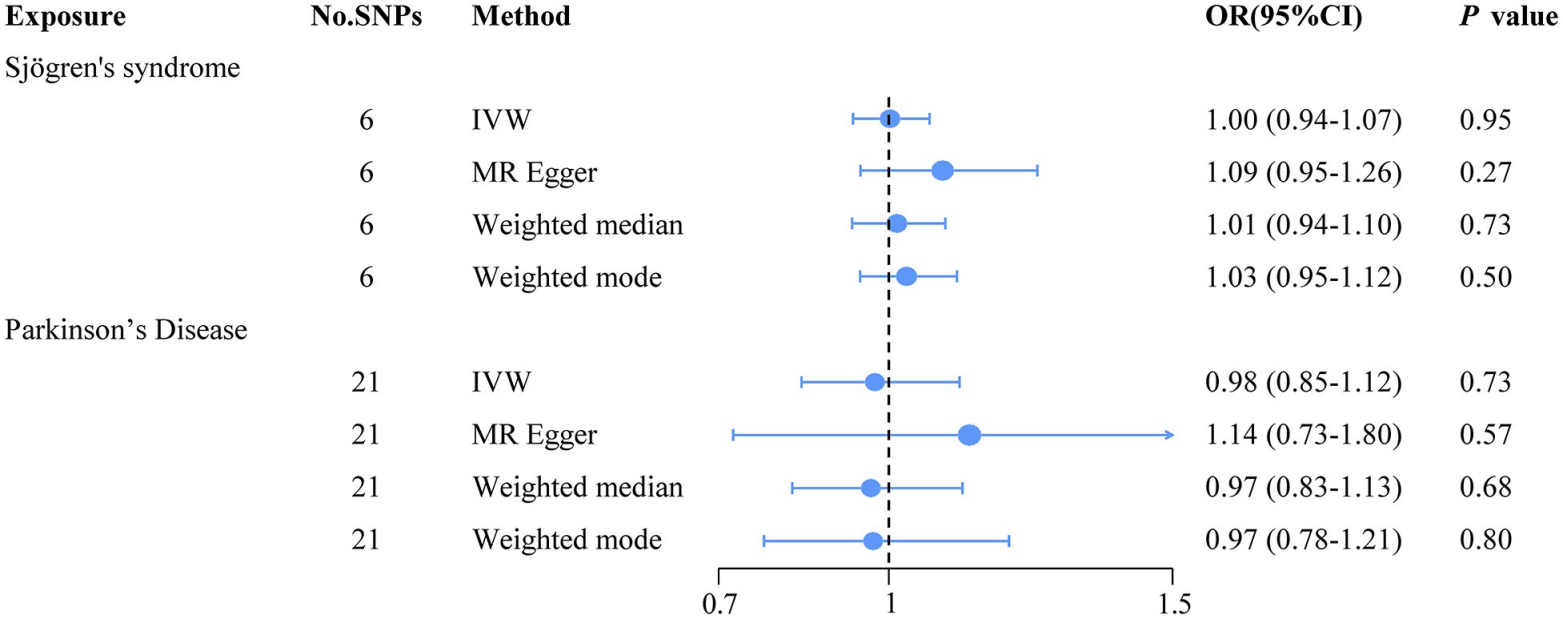
The Mendelian randomization of Sjögren’s syndrome and Parkinson’s Disease.

**Fig 3:**
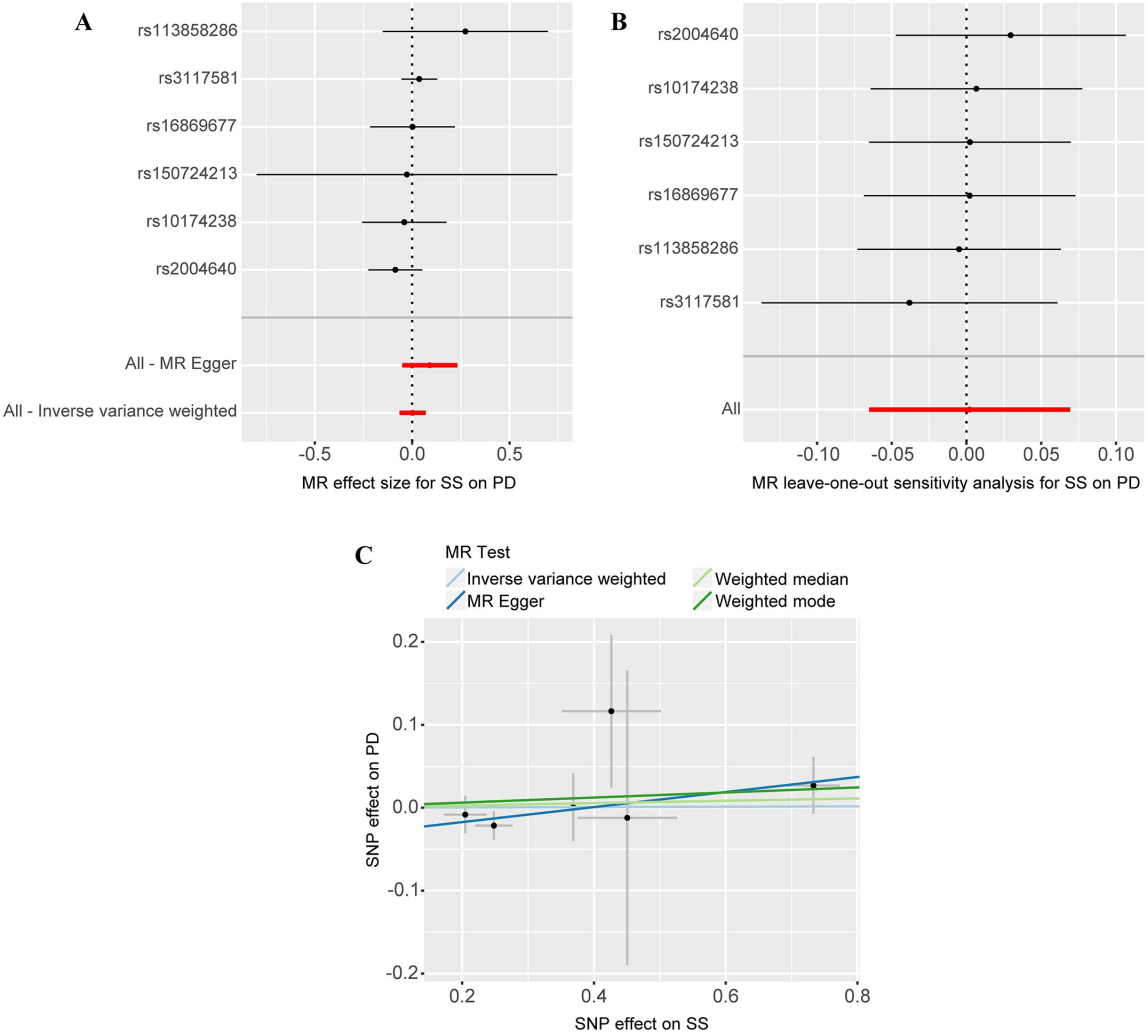
A, B, and C depict the forest plot, leave-one-out analysis, and scatter plot, respectively, illustrating the relationship between Sjögren’s syndrome (SS) and Parkinson’s Disease (PD).

This study included 6 SNPs, and the F statistics for IVs were all greater than 10. These results indicate that the SNPs generally provide ample information for MR studies. S1 Table provides specific information for each SNP. Based on MR estimation, Fig 2 depicts the analysis results. By utilizing the IVW method, we conclude that no significant causal relationship exists between SS and the risk of PD (OR = 1.00, 95% CI = 0.94–1.07, *P* = 0.95). Similarly, the MR-Egger method (OR = 1.09, 95% CI = 0.95–1.26, *P* = 0.27), the weighted median approach (OR = 1.01, 95% CI = 0.94–1.01, *P* = 0.73), and the weighted mode approach (OR = 1.03, 95% CI = 0.95–1.12, *P* = 0.50) yielded similar results (Fig 3A). Cochran’s Q statistic did not reveal significant heterogeneity between the estimated values of the included SNPs (*P* = 0.57). Moreover, leave-one-out analysis validated the stability of the MR estimates (Fig 3B). Consistent with these results, the intercept estimated using MR Egger’s method (intercept = -0.04, *P* = 0.24) and MR-PRESSO tests (global *P* = 0.55) did not provide significant evidence of pleiotropy in our study (Fig 3C). Additionally, we employed PhenoScanner to identify three SNPs that were associated with confounding factors, specifically, rheumatoid arthritis (rs10174238, rs2004640) and diabetes (rs3117581), which have been previously documented as being linked to PD [35, 36]. After excluding these three SNPs, we repeated the MR analysis, which revealed non-existence of a causal association between SS and PD as indicated by the IVW results (OR = 1.06, 95% CI = 0.87–1.27, *P* = 0.58). Furthermore, other supplementary analyses produced consistent findings, while the sensitivity analysis indicated absence of heterogeneity (*P* = 0.52) and pleiotropy (intercept = -0.44, *P* = 0.57). Comprehensive outcomes are elaborated in S3 Table.

### 3.2 Causal effects of PD on SS

**Fig 4:**
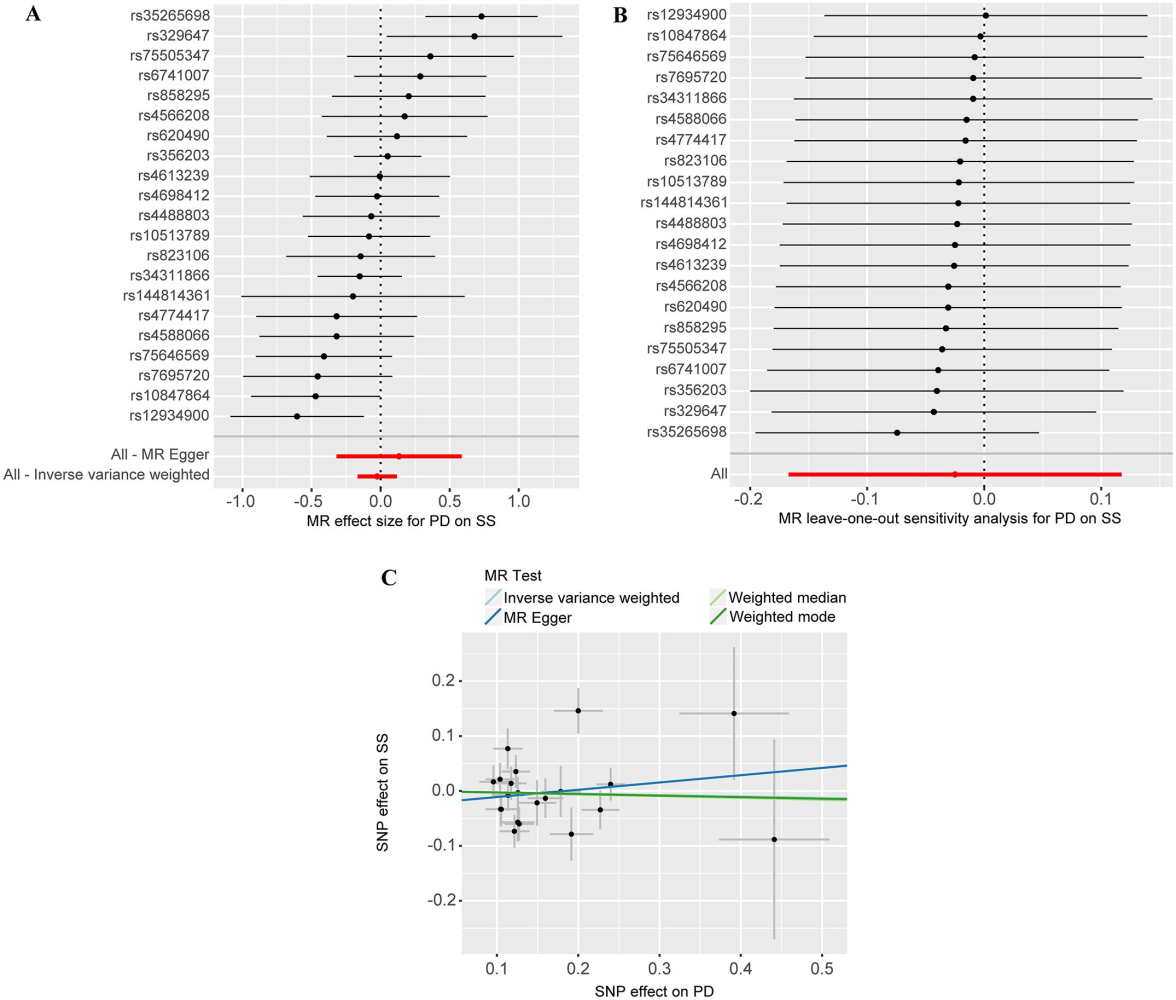
The forest plot (A), leave-one-out analysis (B), and scatter plot (C) examine the impact of Parkinson’s Disease (PD) on Sjögren’s syndrome (SS).

We conducted a reverse MR study to evaluate the presence of a reverse causal relationship between PD (exposure) and SS (outcome). This study comprised a total of 21 SNPs, and the F statistics for the IVs all exceeded a threshold of 10. These findings suggest that the SNPs offer substantial information for conducting MR studies. Detailed information for the IVs is provided in S2 Table. Fig 1 depicts a flowchart illustrating the process of reverse MR. Our study findings did not yield any significant results when utilizing the IVW method (OR = 0.98, 95% CI = 0.85–1.12, *P* = 0.73), MR-Egger method (OR = 1.14, 95% CI = 0.73–1.80, *P* = 0.57), weighted median method (OR = 0.97, 95% CI = 0.83–1.13, *P* = 0.68), and weighted model method (OR = 0.97, 95% CI = 0.78–1.21, *P* = 0.80), as presented in Figs 2 and 4A. Additionally, the Cochran’s Q statistic indicated the presence of heterogeneity (*P* = 0.01). Leave-one-out analysis demonstrated that specific SNPs did not significantly influence the MR estimates, as observed in Fig 4B. The intercept for the MR-Egger method was estimated to be -0.02 (*P* = 0.48), and the MR-PRESSO global tests resulted in a *P*-value of 0.24, indicating the absence of apparent horizontal pleiotropy, as demonstrated in Fig 4C. In PhenoScanner, one SNPs (rs35265698) was identified as being associated with rheumatoid arthritis, which has previously been reported to be related to SS [37]. After excluding SNPs that are associated with confounding factors, the IVW analysis results indicate a lack of significant causal association between PD and SS (OR = 0.93, 95% CI = 0.82–1.05, *P* = 0.23), which is consistent with the results obtained from several other supplementary analysis methods. Sensitivity analysis did not detect any heterogeneity (*P* = 0.14) or pleiotropy (intercept = -0.01, *P* = 0.76). The detailed results can be found in S3 Table.

## 4. Discussion

To the best of our understanding, this research represents the initial utilization of a two-sample MR approach to investigate the potential causal association between SS and PD in populations of European ancestry. The outcomes of this study fail to furnish substantiation for a reciprocal genetic connection between these two disorders. Nevertheless, caution should be exercised when interpreting these results due to the limited number of available GWAS conducted on SS and PD thus far.

Our MR research findings align with previous observational studies, indicating an absence of a bidirectional causal relationship between SS and PD. Li et al. [18] conducted a study in Sweden involving 310,522 patients with autoimmune diseases, aiming to investigate the relationship between 31 autoimmune diseases and PD. The follow-up results for patients with SS displayed no heightened risk of developing PD (Standardized incidence ratios = 2.01, 95% CI = 0.63–4.72). Similarly, a case-control study conducted in Denmark, including 13,695 PD patients and 68,445 control individuals, also indicated no significant correlation between the risk of PD and SS-diagnosed patients (OR = 0.96, 95% CI = 0.85–1.08) [19]. However, it is worth noting that previous observational studies have reported a correlation between SS and PD, contradicting our findings. For instance, a cohort study carried out in Taiwan, involving 50,560 controls and 12,640 SS patients, identified a significant increase in the incidence of PD among SS patients (adjusted hazard ratio = 1.23, 95% CI = 1.16–1.30) [38]. The results of a case-control study by Wu et al. [16] indicated a significantly heightened risk of developing PD in SS patients (OR = 1.37, 95% CI = 1.15–1.65). Furthermore, a recent meta-analysis also reported an increased risk of PD in SS patients (OR = 1.61, 95% CI = 1.24–2.09), while highlighting heterogeneity in the research results (I^2^ = 61.9%) [17]. Although these studies suggest a correlation between SS and PD, it is important to note that observational research cannot establish causality, and the observed association in the real world may be influenced by population differences and residual confounding factors.

The interpretation of the finding that there is no significant genetic causal relationship between SS and PD should be approached with caution. It is important to note that rheumatoid arthritis serves as a common risk factor for both SS and PD [36, 37], and as a result, the observed correlation between SS and PD in observational studies may be confounded by this shared risk factor. Furthermore, inflammation plays a crucial role in the development of cardiovascular diseases [39], and it is worth mentioning that SS patients often have comorbid cardiovascular conditions [40], which are also prevalent in PD patients [41]. Consequently, it is plausible that the link between SS and PD may be attributed to certain shared inflammatory pathways, as the increase of pro-inflammatory factors in the brain can contribute to the progression of PD and neurodegeneration [8]. Moreover, the amplified risk of developing PD in SS patients can potentially be explained by discrepancies in lifestyle and nutrition. For instance, it is common for SS patients to experience vitamin D deficiency [42], and research indicates that vitamin D exerts a protective effect against PD [43]. Therefore, the heightened risk of PD in SS patients may be connected to the deficiency of vitamin D. Another factor to consider is that SS patients often require ongoing treatment and monitoring of disease progression. This heightened healthcare involvement may contribute to the earlier detection and diagnosis of PD compared to the control population [44], thus influencing the association between SS and PD risk in real-world settings. Additionally, it is important to acknowledge that SS patients often require long-term use of immunosuppressants, and PD is also influenced by immune system-related factors. Consequently, there is a possibility of bias in the results. According to Ju et al., long-term use of hydroxychloroquine in SS patients was found to increase the risk of PD when compared to the control group (HR = 1.46, 95% CI = 1.34–1.59) [38]. Other observational studies do not report on this crucial factor, and further investigation into the effects of immunosuppressants on PD could enhance the clinical management of these patients. Furthermore, studies have revealed that patients with SS and concurrent PD may manifest heightened levels of anti-β2-glycoprotein-I and anti-cardiolipin antibodies [45]. Consequently, there may be concurrent immune dysregulation in both SS and PD, giving rise to a reported correlation between these two phenotypes in certain observational studies. It is worth noting that our discussion regarding the relationship between SS and PD is confined to a genetic causality perspective. The correlation between SS and PD encompasses intricate underlying mechanisms that necessitate further comprehensive research. Subsequent studies could investigate the association between SS and PD considering inflammation, immunity, and immunosuppressants. Furthermore, the interplay between environmental and genetic factors plays a significant role in disease development, and a more comprehensive understanding of the relationship between SS and PD can be achieved by considering these aspects.

Our research possesses several notable strengths. Firstly, the implementation of MR design serves to reduce residual confounding and various biases, thereby augmenting the strength of our causal inference. Furthermore, the incorporation of bidirectional MR analysis facilitates the examination of causality between SS and PD in both directions. Additionally, we ensured the evaluation of these associations within two distinct populations, thereby enhancing the reliability of our findings. Additionally, we performed multiple sensitivity analyses, enabling us to obtain consistent estimates from various models. This finding further enhanced our confidence in the established correlations. Additionally, we integrated the latest GWAS of SS and PD among individuals of European descent, ensuring adequate statistical strength to assess the potential causal connection between SS and PD, while concurrently mitigating the impact of population stratification.

However, our MR study has certain limitations that must be acknowledged. Firstly, the inclusion of GWAS databases exclusively derived from European populations in our study raises the question of whether this causal association remains significant in other populations, necessitating further investigation. Secondly, the unavailability of comprehensive demographic and clinical data on the participants prevented us from conducting subgroup analyses. Thirdly, our MR study focused solely on assessing the effects of lifetime exposure, potentially leading to overestimation in real-world scenarios where effective interventions are implemented.

## 5. Conclusion

In summary, the findings of our MR study do not currently provide support for the hypothesis positing a genetic causal association between SS and PD. Furthermore, our investigation has not yielded conclusive evidence substantiating a causal connection between PD and the susceptibility to SS. To fully validate our research outcomes, additional comprehensive mechanism studies and subsequent investigations involving larger sample sizes and multiple research centers are imperative. It is crucial to acknowledge that our MR analysis exclusively focuses on genetic hereditary relationships and does not elucidate other potential causal associations.

## Supporting information

S1 Table Detailed information of instrumental variables used in MR analyses (Causal effects of SS on PD). (XLSX)

S2 Table Detailed information of instrumental variables used in MR analyses (Causal effects of PD on SS). (XLSX)

S3 Table Additional Mendelian randomization analysis by eliminating confounding factors. (XLSX)

## References

[1] Choudhry HS, Hosseini S, Choudhry HS, Fatahzadeh M, Khianey R, Dastjerdi MH. Updates in diagnostics, treatments, and correlations between oral and ocular manifestations of Sjogren’s syndrome. Ocul Surf. 2022;26:75–87. doi: 10.1016/j.jtos.2022.08.001 35961534

[2] Wang B, Chen S, Zheng Q, Li Y, Zhang X, Xuan J, et al. Early diagnosis and treatment for Sjögren’s syndrome: current challenges, redefined disease stages and future prospects. J Autoimmun. 2021;117:102590. doi: 10.1016/j.jaut.2020.102590 33310686

[3] Goules AV, Tzioufas AG. Primary Sjögren’s syndrome: clinical phenotypes, outcome and the development of biomarkers. Immunol Res. 2017;65(1):331–44. doi: 10.1007/s12026-016-8844-4 27444892

[4] McCoy SS, Woodham M, Bunya VY, Saldanha IJ, Akpek EK, Makara MA, et al. A comprehensive overview of living with Sjögren’s: results of a National Sjögren’s Foundation survey. Clin Rheumatol. 2022;41(7):2071–8. doi: 10.1007/s10067-022-06119-w 35257256 PMC9610846

[5] Goulabchand R, Castille E, Navucet S, Etchecopar-Etchart D, Matos A, Maria A, et al. The interplay between cognition, depression, anxiety, and sleep in primary Sjogren’s syndrome patients. Sci Rep-Uk. 2022;12(1):13176. doi: 10.1038/s41598-022-17354-1 35915312 PMC9343365

[6] Thalayasingam N, Baldwin K, Judd C, Ng WF. New developments in Sjogren’s syndrome. Rheumatology. 2021;60(Suppl 6):vi53–61. doi: 10.1093/rheumatology/keab466 34951923 PMC8709567

[7] Sen T, Saha P, Gupta R, Foley LM, Jiang T, Abakumova OS, et al. Aberrant ER Stress Induced Neuronal-IFNβ Elicits White Matter Injury Due to Microglial Activation and T-Cell Infiltration after TBI. J Neurosci. 2020;40(2):424–46. doi: 10.1523/JNEUROSCI.0718-19.2019 31694961 PMC6948950

[8] Appenzeller S, Andrade SO, Bombini MF, Sepresse SR, Reis F, França MJ. Neuropsychiatric manifestations in primary Sjogren syndrome. Expert Rev Clin Immu. 2022;18(10):1071–81. doi: 10.1080/1744666X.2022.2117159 36001085

[9] Tolosa E, Garrido A, Scholz SW, Poewe W. Challenges in the diagnosis of Parkinson’s disease. Lancet Neurol. 2021;20(5):385–97. doi: 10.1016/S1474-4422(21)00030-2 33894193 PMC8185633

[10] Zhang X, Gao F, Wang D, Li C, Fu Y, He W, et al. Tau Pathology in Parkinson’s Disease. Front Neurol. 2018;9:809. doi: 10.3389/fneur.2018.00809 30333786 PMC6176019

[11] Li J, Zhu BF, Gu ZQ, Zhang H, Mei SS, Ji SZ, et al. Musculoskeletal Pain in Parkinson’s Disease. Front Neurol. 2021;12:756538. doi: 10.3389/fneur.2021.756538 35126283 PMC8813739

[12] Global, regional, and national burden of Parkinson’s disease, 1990–2016: a systematic analysis for the Global Burden of Disease Study 2016. Lancet Neurol. 2018;17(11):939–53. doi: 10.1016/S1474-4422(18)30295-3 30287051 PMC6191528

[13] Tansey MG, Wallings RL, Houser MC, Herrick MK, Keating CE, Joers V. Inflammation and immune dysfunction in Parkinson disease. Nat Rev Immunol. 2022;22(11):657–73. doi: 10.1038/s41577-022-00684-6 35246670 PMC8895080

[14] Jiang T, Li G, Xu J, Gao S, Chen X. The Challenge of the Pathogenesis of Parkinson’s Disease: Is Autoimmunity the Culprit? Front Immunol. 2018;9:2047. doi: 10.3389/fimmu.2018.02047 30319601 PMC6170625

[15] Hsu HC, Hou TY, Lin TM, Chang YS, Chen WS, Kuo PI, et al. Higher risk of Parkinson disease in patients with primary Sjögren’s syndrome. Clin Rheumatol. 2020;39(10):2999–3007. doi: 10.1007/s10067-020-05053-z 32240432

[16] Wu MC, Xu X, Chen SM, Tyan YS, Chiou JY, Wang YH, et al. Impact of Sjogren’s syndrome on Parkinson’s disease: A nationwide case-control study. Plos One. 2017;12(7):e175836. doi: 10.1371/journal.pone.0175836 28704397 PMC5509109

[17] Li M, Wan J, Xu Z, Tang B. The association between Parkinson’s disease and autoimmune diseases: A systematic review and meta-analysis. Front Immunol. 2023;14:1103053. doi: 10.3389/fimmu.2023.1103053 36761731 PMC9905134

[18] Li X, Sundquist J, Sundquist K. Subsequent risks of Parkinson disease in patients with autoimmune and related disorders: a nationwide epidemiological study from Sweden. Neurodegener Dis. 2012;10(1–4):277–84. doi: 10.1159/000333222 22205172

[19] Rugbjerg K, Friis S, Ritz B, Schernhammer ES, Korbo L, Olsen JH. Autoimmune disease and risk for Parkinson disease: a population-based case-control study. Neurology. 2009;73(18):1462–8. doi: 10.1212/WNL.0b013e3181c06635 19776374 PMC2779008

[20] Vansteelandt S. Estimating direct effects in cohort and case-control studies. Epidemiology. 2009;20(6):851–60. doi: 10.1097/EDE.0b013e3181b6f4c9 19806060

[21] Birney E. Mendelian Randomization. Csh Perspect Med. 2022;12(4). doi: 10.1101/cshperspect.a041302 34872952 PMC9121891

[22] Cifuentes M, Grandont L, Moore G, Chèvre AM, Jenczewski E. Genetic regulation of meiosis in polyploid species: new insights into an old question. New Phytol. 2010;186(1):29–36. doi: 10.1111/j.1469-8137.2009.03084.x 19912546

[23] Sekula P, F Del G M, Pattaro C, Köttgen A. Mendelian Randomization as an Approach to Assess Causality Using Observational Data. J Am Soc Nephrol. 2016;27(11):3253–65. doi: 10.1681/ASN.2016010098 27486138 PMC5084898

[24] Bowden J, Holmes MV. Meta-analysis and Mendelian randomization: A review. Res Synth Methods. 2019;10(4):486–96. doi: 10.1002/jrsm.1346 30861319 PMC6973275

[25] Kurki MI, Karjalainen J, Palta P, Sipilä TP, Kristiansson K, Donner KM, et al. FinnGen provides genetic insights from a well-phenotyped isolated population. Nature. 2023;613(7944):508–18. doi: 10.1038/s41586-022-05473-8 36653562 PMC9849126

[26] Ten Years of the International Parkinson Disease Genomics Consortium: Progress and Next Steps. J Parkinson Dis. 2020;10(1):19–30. doi: 10.3233/JPD-191854 31815703 PMC7029327

[27] Burgess S, Thompson SG. Avoiding bias from weak instruments in Mendelian randomization studies. Int J Epidemiol. 2011;40(3):755–64. doi: 10.1093/ije/dyr036 21414999

[28] Burgess S, Small DS, Thompson SG. A review of instrumental variable estimators for Mendelian randomization. Stat Methods Med Res. 2017;26(5):2333–55. doi: 10.1177/0962280215597579 26282889 PMC5642006

[29] Burgess S, Butterworth A, Thompson SG. Mendelian randomization analysis with multiple genetic variants using summarized data. Genet Epidemiol. 2013;37(7):658–65. doi: 10.1002/gepi.21758 24114802 PMC4377079

[30] Bowden J, Davey SG, Burgess S. Mendelian randomization with invalid instruments: effect estimation and bias detection through Egger regression. Int J Epidemiol. 2015;44(2):512–25. doi: 10.1093/ije/dyv080 26050253 PMC4469799

[31] Bowden J, Davey SG, Haycock PC, Burgess S. Consistent Estimation in Mendelian Randomization with Some Invalid Instruments Using a Weighted Median Estimator. Genet Epidemiol. 2016;40(4):304–14. doi: 10.1002/gepi.21965 27061298 PMC4849733

[32] Hemani G, Zheng J, Elsworth B, Wade KH, Haberland V, Baird D, et al. The MR-Base platform supports systematic causal inference across the human phenome. Elife. 2018;7. doi: 10.7554/eLife.34408 29846171 PMC5976434

[33] Verbanck M, Chen CY, Neale B, Do R. Detection of widespread horizontal pleiotropy in causal relationships inferred from Mendelian randomization between complex traits and diseases. Nat Genet. 2018;50(5):693–8. doi: 10.1038/s41588-018-0099-7 29686387 PMC6083837

[34] Sanderson E, Davey SG, Windmeijer F, Bowden J. An examination of multivariable Mendelian randomization in the single-sample and two-sample summary data settings. Int J Epidemiol. 2019;48(3):713–27. doi: 10.1093/ije/dyy262 30535378 PMC6734942

[35] Aune D, Schlesinger S, Mahamat-Saleh Y, Zheng B, Udeh-Momoh CT, Middleton LT. Diabetes mellitus, prediabetes and the risk of Parkinson’s disease: a systematic review and meta-analysis of 15 cohort studies with 29.9 million participants and 86,345 cases. Eur J Epidemiol. 2023;38(6):591–604. doi: 10.1007/s10654-023-00970-0 37185794 PMC10232631

[36] Bacelis J, Compagno M, George S, Pospisilik JA, Brundin P, Naluai ÅT, et al. Decreased Risk of Parkinson’s Disease After Rheumatoid Arthritis Diagnosis: A Nested Case-Control Study with Matched Cases and Controls. J Parkinson Dis. 2021;11(2):821–32. doi: 10.3233/JPD-202418 33682730 PMC8150472

[37] Kim H, Cho SK, Kim HW, Han J, Kim Y, Hwang KG, et al. The Prevalence of Sjögren’s Syndrome in Rheumatoid Arthritis Patients and Their Clinical Features. J Korean Med Sci. 2020;35(45):e369. doi: 10.3346/jkms.2020.35.e369 33230982 PMC7683240

[38] Ju UH, Liu FC, Lin CS, Huang WY, Lin TY, Shen CH, et al. Risk of Parkinson disease in Sjögren syndrome administered ineffective immunosuppressant therapies: A nationwide population-based study. Medicine. 2019;98(14):e14984. doi: 10.1097/MD.0000000000014984 30946325 PMC6455855

[39] Lawler PR, Bhatt DL, Godoy LC, Lüscher TF, Bonow RO, Verma S, et al. Targeting cardiovascular inflammation: next steps in clinical translation. Eur Heart J. 2021;42(1):113–31. doi: 10.1093/eurheartj/ehaa099 32176778

[40] Casian M, Jurcut C, Dima A, Mihai A, Stanciu S, Jurcut R. Cardiovascular Disease in Primary Sjögren’s Syndrome: Raising Clinicians’ Awareness. Front Immunol. 2022;13:865373. doi: 10.3389/fimmu.2022.865373 35757738 PMC9219550

[41] Potashkin J, Huang X, Becker C, Chen H, Foltynie T, Marras C. Understanding the links between cardiovascular disease and Parkinson’s disease. Movement Disord. 2020;35(1):55–74. doi: 10.1002/mds.27836 31483535 PMC6981000

[42] Erten Ş, Şahin A, Altunoğlu A, Gemcioğlu E, Koca C. Comparison of plasma vitamin D levels in patients with Sjögren’s syndrome and healthy subjects. Int J Rheum Dis. 2015;18(1):70–5. doi: 10.1111/1756-185X.12298 24467766

[43] Rimmelzwaan LM, van Schoor NM, Lips P, Berendse HW, Eekhoff EM. Systematic Review of the Relationship between Vitamin D and Parkinson’s Disease. J Parkinson Dis. 2016;6(1):29–37. doi: 10.3233/JPD-150615 26756741 PMC4927872

[44] Wang T, Shi C, Luo H, Zheng H, Fan L, Tang M, et al. Neuroinflammation in Parkinson’s Disease: Triggers, Mechanisms, and Immunotherapies. Neuroscientist. 2022;28(4):364–81. doi: 10.1177/1073858421991066 33576313

[45] Hassin-Baer S, Levy Y, Langevitz P, Nakar S, Ehrenfeld M. Anti-beta2-glycoprotein I in Sjogren’s syndrome is associated with parkinsonism. Clin Rheumatol. 2007;26(5):743–7. doi: 10.1007/s10067-006-0398-8 17057945

